# Quantifying the Adaptive Cycle

**DOI:** 10.1371/journal.pone.0146053

**Published:** 2015-12-30

**Authors:** David G. Angeler, Craig R. Allen, Ahjond S. Garmestani, Lance H. Gunderson, Olle Hjerne, Monika Winder

**Affiliations:** 1 Stockholm University, Department of Ecology, Evolution and Plant Sciences, SE- 106 91, Stockholm, Sweden; 2 Swedish University of Agricultural Sciences, Department of Aquatic Sciences and Assessment, Box 7050, SE- 750 07, Uppsala, Sweden; 3 U.S. Geological Survey—Nebraska Cooperative Fish & Wildlife Research Unit, University of Nebraska, Lincoln, NE, 68583, United States of America; 4 U.S. Environmental Protection Agency, National Risk Management Research Laboratory, Cincinnati, OH, 45268, United States of America; 5 Department of Environmental Sciences, Emory University, Atlanta, Georgia, 30322, United States of America; University of Florida, UNITED STATES

## Abstract

The adaptive cycle was proposed as a conceptual model to portray patterns of change in complex systems. Despite the model having potential for elucidating change across systems, it has been used mainly as a metaphor, describing system dynamics qualitatively. We use a quantitative approach for testing premises (reorganisation, conservatism, adaptation) in the adaptive cycle, using Baltic Sea phytoplankton communities as an example of such complex system dynamics. Phytoplankton organizes in recurring spring and summer blooms, a well-established paradigm in planktology and succession theory, with characteristic temporal trajectories during blooms that may be consistent with adaptive cycle phases. We used long-term (1994–2011) data and multivariate analysis of community structure to assess key components of the adaptive cycle. Specifically, we tested predictions about: reorganisation: spring and summer blooms comprise distinct community states; conservatism: community trajectories during individual adaptive cycles are conservative; and adaptation: phytoplankton species during blooms change in the long term. All predictions were supported by our analyses. Results suggest that traditional ecological paradigms such as phytoplankton successional models have potential for moving the adaptive cycle from a metaphor to a framework that can improve our understanding how complex systems organize and reorganize following collapse. Quantifying reorganization, conservatism and adaptation provides opportunities to cope with the intricacies and uncertainties associated with fast ecological change, driven by shifting system controls. Ultimately, combining traditional ecological paradigms with heuristics of complex system dynamics using quantitative approaches may help refine ecological theory and improve our understanding of the resilience of ecosystems.

## Introduction

Structural and functional properties and processes of complex adaptive systems are dynamic, with periods of growth, destruction and decay that vary across scales of space and time [1]. Within the ecological sciences, succession of forests through different stages over centuries [2] or the seasonal dynamics of plankton [3] are examples of constant change and renewal, and have become well-established paradigms in ecology. Within the social systems, the rise and fall of ancient societies [4] and changes in current western societies, driven by industrial and technological advances [5], are analogous examples of dynamic system change.

**Fig 1:**
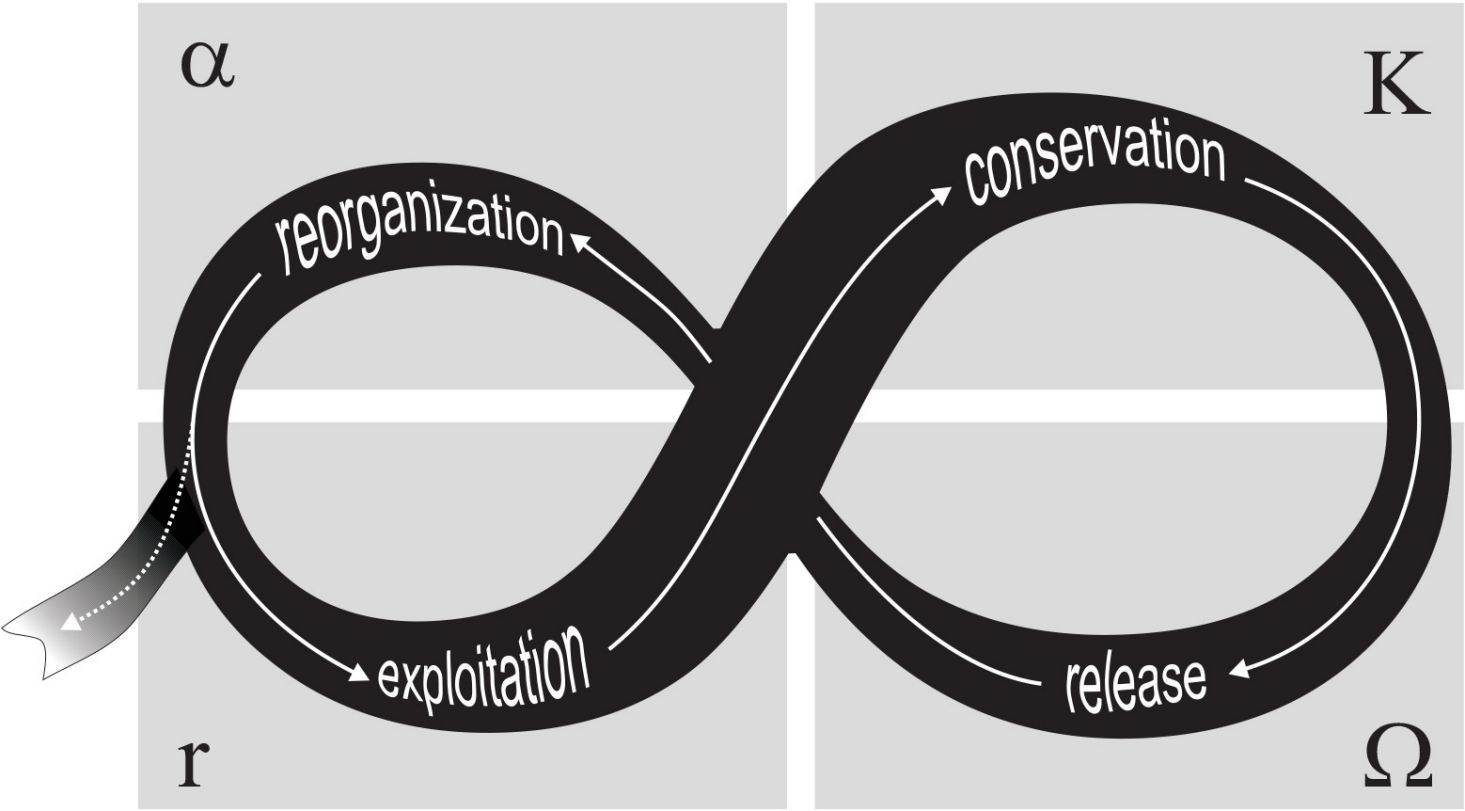
Schematic description of the adaptive cycle. It shows transitioning between four phases (α, r, K, Ω) within a specific regime (full white arrows) and the potential to change to a new regime in the reorganisation phase (dotted white arrow). Modified from Gunderson and Holling (2002).

Comparing patterns of change across complex systems has led to the development of a model that accounts for observed dynamics, while simplifying the complexity inherent in ecological and social systems, referred to as an adaptive cycle [6–7]. Change is characterized in four phases: (1) an r-phase, which consists of rapid exploitation of resources and therefore fast growth, (2) a K-phase comprising a steady-state period and conservation of resources and structure, (3) an Ω-phase when the system collapses, releasing accumulated energy and resources, and (4) an α-phase when the system reorganizes, either into a configuration with similar or different properties; that is, when the system either reorganizes within the same basin of attraction or when it undergoes a regime shift (Fig 1). From a systems perspective, these four phases emphasize change in the organization of complex systems as a result of shifting feedbacks and controls. This capacity to organize in response to disturbances has therefore implications for the resilience of complex systems [8].

The conceptual linkages between system attributes (e.g., species composition and their functional roles), processes and resilience have made the adaptive cycle a valuable tool in the analysis and management of social and ecological systems in the current period of rapid environmental and social-ecological change [9]. However, the adaptive cycle has been used primarily in a qualitative context examining societal responses to environmental, climate and economic changes [10–12], human behaviour [13,14], and social-ecological systems dynamics [15,16]. Ecological studies of the adaptive cycle are scant and merely descriptive [17].

As a heuristic, the adaptive cycle can help envision the organization of seemingly complex dynamics in ecosystems, linked social–ecological systems and governance [1]. For the adaptive cycle model to develop beyond a conceptual framework for envisioning complex dynamics and to provide useful management guidance, explicit tests of the underlying premises are required. The adaptive cycle covers many facets of complex system dynamics including reorganization, conservatism and adaptation. In this paper we use phytoplankton succession to test these facets of the adaptive cycle. We define reorganization as a substantial change in phytoplankton community structure between spring and summer blooms, which occur during seasonal succession cycles [3]. Conservatism refers to community trajectories during individual adaptive cycles that may include community build-up, saturation, and collapse phases independent of the taxonomic composition of blooms. Adaptation accounts for taxonomic turnover within inter-annually recurring (conservative) blooms in the long-term, which may be modulated by changing abiotic and biotic conditions. Assessing these facets in complex systems dynamics has been challenging. Most quantitative studies use complex modelling and simulation approaches for assessing complex adaptive systems [18–20]. The adaptive cycle model provides opportunities to assess these facets in real systems using statistical tools common in ecology. An understanding of reorganization, conservatism and adaptation may refine current theories regarding system dynamics (e.g. resilience theory) and may provide management guidance and decision support.

**Fig 2:**
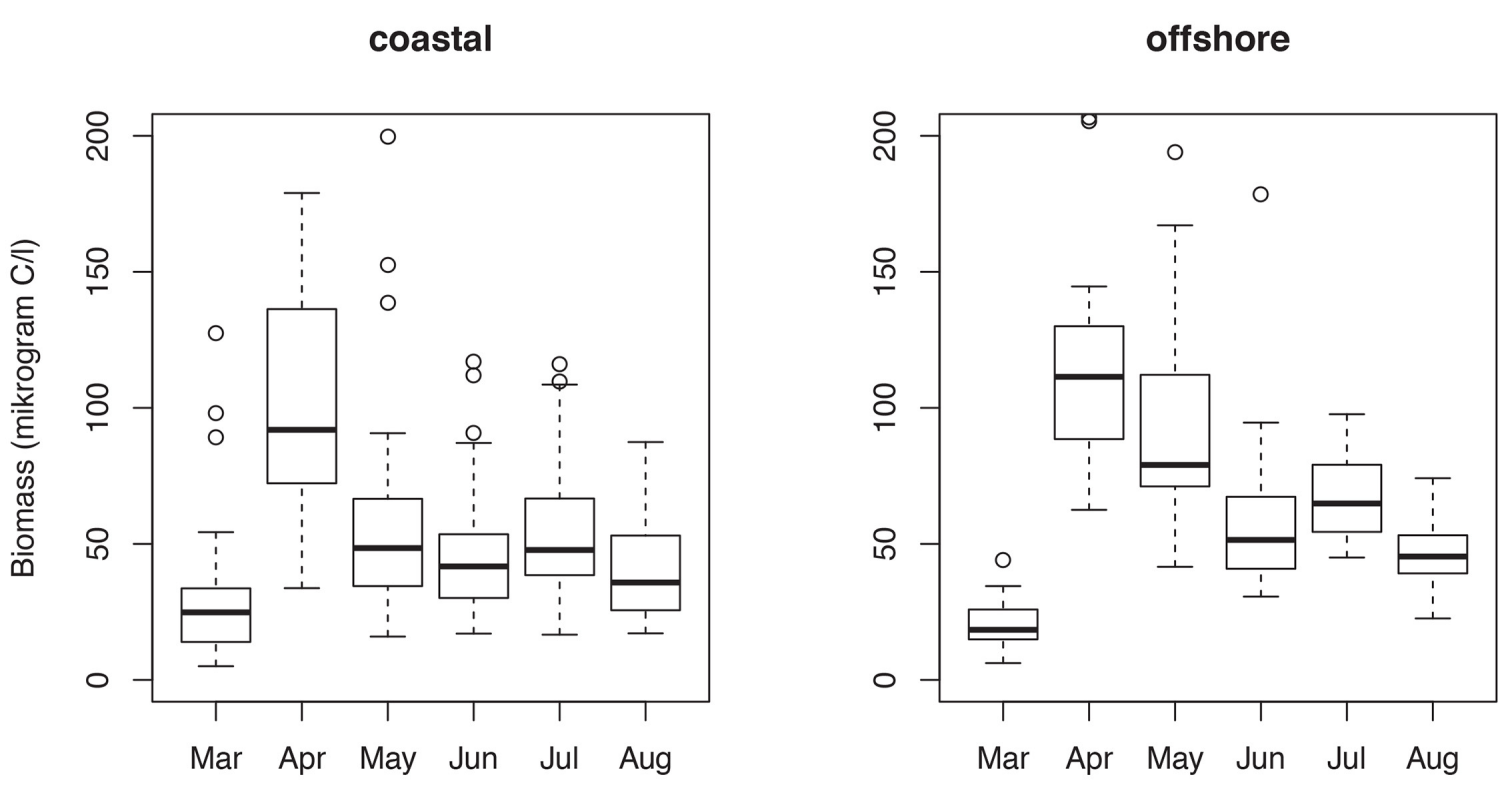
Phytoplankton biomass. Box plots showing phytoplankton biomass (microgram C L^-1^) between March and August for the period 1994–2011 at two sites (coastal, offshore) in the Baltic Sea. Two periods with peak biomass in April and July occur at both sites, and characterize distinct spring and summer phytoplankton blooms in the Baltic Sea.

We used a quantitative approach to study long-term dynamics (1994–2011) of phytoplankton communities in the Baltic Sea. Phytoplankton is a useful model for studying adaptive cycles for the following reasons. Communities are species rich and dynamics show complex adaptive system behaviour, modulated through feedbacks that arise between interacting biological (competition and top-down effects resulting from zooplankton grazing and trophic cascades) and abiotic (nutrients, temperature, stratification) factors [1,21,22]. These dynamics have been widely studied in planktology and succession theory, and have become well-established paradigms in ecology [2,3,22]. These paradigms are useful for an assessment of adaptive cycles from a structural perspective through analyses of the taxonomic composition of communities. Phytoplankton communities demonstrate seasonal patterns with groups of species developing into spring and summer blooms [22,23], although these patterns can vary between ecosystems [24]. Given this context dependence, it is important to define the temporal window of bloom dynamics to delineate the adaptive cycles for the study system. Specifically in the Baltic Sea, spring blooms typically occur between March and May, while summer blooms develop from June to August (Fig 2). These blooming periods are stable and recur annually, providing an opportunity to assess predictions that follow from the characterization of these dynamics as consistent with adaptive cycles.

In the Baltic Sea, the spring bloom is a response to seasonal increases in solar radiation and temperature, and possibly stratification caused by vertical thermal and salinity gradients following winter mixing. The bloom is initially comprised of fast-growing diatoms, followed by slower-growing dinoflagellates. Spring blooms are characterized by high phytoplankton biomass. As nutrients are depleted and temperatures increase, spring blooms collapse and the phytoplankton community reorganizes into a summer bloom, during which many inedible taxa, including cyanobacteria and flagellates, dominate. This is partly due to a reconfiguration of the food web in summer that results in altered biotic feedbacks whereby zooplankton grazing and trophic cascades influence community dynamics of phytoplankton [3,22]. In short, system extrinsic (environmental variables) and intrinsic (biological interaction) factors interact to mediate community dynamics within and between spring and summer blooms, thereby influencing the adaptive cycle-type behavior of phytoplankton. Furthermore, phytoplankton in the Baltic Sea undergo changes both in community composition and bloom phenology in the long term [25–27], a consequence of anthropogenic environmental change, including eutrophication and overfishing that alter abiotic and biotic controls in the system [28–30]. However, complex cause-effect chains between winter temperature and nutrient dynamics influence feedbacks in the Baltic Sea [31]. The complex interaction between natural and anthropogenic factors leads to process in the phytoplankton community that filter out species from the community that are adapted to the resulting environmental conditions; also community responses to these changes differ between spring and summer [31].

The seasonally recurring spring and summer blooms together with longer-term environmental changes that affect phytoplankton dynamics allow for testing specific predictions regarding reorganization while maintaining adaptability in this specific complex system. We use observations made in phytoplankton successional studies and adopt them to the adaptive cycle model testing the following predictions:

1. Phytoplankton operate in different community states or adaptive cycles given distinct abiotic and biotic conditions between spring and summer blooms.
2. During community trajectories within blooms, distinct phases of the adaptive cycle, reminiscent of community build-up, saturation, and collapse, are evident and these patterns are preserved during recurrent blooms. These community trajectories within spring and summer blooms are therefore expected to be conservative.
3. Changes of ecological baselines due to environmental change (e.g. climate warming, nutrient patterns, foodweb change) modulates phytoplankton responses that affect the spring and summer blooms differentially. That is, despite conservative seasonal community trajectories within blooms, environmental and biological filtering processes select for sets of species adapted to prevailing ecological conditions in the Baltic Sea in the long term.

## Material and Methods

### Ethics Statement

All field sampling and laboratory analyses reported in this study are part of the Baltic Sea Monitoring Program and are approved by the Swedish Agency for Marine and Water Management (HaV). Data used in this study are available through the Dryad Digital Repository (http://dx.doi.org/10.5061/dryad.8hj8t) or the Swedish Meteorological and Hydrological Institute (SMHI). It is confirmed that the field studies did not involve endangered or protected species.

### Sites and sampling

Standardized sampling and analysis protocols were used throughout the study. Water quality (temperature, phosphate, dissolved inorganic nitrogen, Secchi depth, salinity, mixed layer depth) and phytoplankton were assessed at the coastal station B1 near Askö (58°48’ N, 17°38’ E, 40 m deep) and from the offshore station BY31 at Landsort Deep (58°35.90’ N, 18°14.21’ E, 459 m deep), in the NW Baltic Proper, the southern part of the Baltic Sea. We here used data collected approximately fortnightly and spanning 1994–2011 for spring (March to May) and summer (June to August) for the coastal and offshore station.

Secchi depth was measured by lowering a white 250 mm disc into the water until it could not be seen through a water scope. Temperature, salinity, dissolved inorganic nitrogen (DIN), phosphate and Chlorophyll *a* were measured every fifth meter and we estimated average values for the upper 20 m (for chemical analysis and further details see [32]). For calculating mixed layer depth (MLD), we estimated vertical profiles of water density from temperature, salinity and depth data from a CTD probe. We defined the MLD as the depth where the density was 0.15 kg·m^-3^ higher than at 1.5 m depth.

Phytoplankton samples were taken as integrated samples with a sampling hose (inner diameter 19 mm) from 0 to 20 m and preserved with acid Lugol’s solution [33]. Phytoplankton (> 2 μm) were counted after sedimentation in 10- or 25-mL chambers using an inverted microscope with phase contrast and cells were measured and size classed according to the methods described in the HELCOM guidelines [34]. Species-specific, size-classed cell volumes were used to calculate the biovolume of each species, using the recommendations by Olenina et al. [35] and the related standard volumes (http://www.ices.dk/marine-data/vocabularies/Documents/PEG_BVOL.zip).

### Statistical analyses

#### Water quality

We calculated a seasonal average and standard deviations of water quality variables for each season and tracked their patterns in the coastal and offshore site over the study period (1994–2011). This showed that the abiotic environment differs between summer and spring. A similar analysis was carried out for Chlorophyll *a*, a measure of phytoplankton biomass. We also used nonmetric multidimensional scaling ordinations (NMDS) to complement the analysis of univariate patterns with a multivariate approach. Water quality data used in the analysis (excluding Chlorophyll *a*) were standardized and converted into a Euclidean distance matrix before analysis. The NMDS result was based on 999 re-runs to achieve convergence of the ordination solution, and the resulting stress value (0.03) indicated a reliable solution [36].

#### Phytoplankton

We used NMDS to show the adaptive cycle behaviour of phytoplankton communities during spring and summer. The ordinations were based on Bray–Curtis dissimilarity matrices, which were calculated from square-root transformed phytoplankton biovolume data for each sampling event (n = 7–9 between March and May [spring], n = 6–7 between June and August [summer]) during each season. Because an analysis including the whole data set resulted in an unreliable ordination (stress > 0.2; [36]), each site and season was analyzed separately using all years in NMDS analyses. Separate analyses were considered appropriate for visually confirming the quantitative approach based on permutational multivariate ANOVA (below), although we acknowledge that this division limits an assessment of the transition (or re-organization) phase between spring and summer blooms. Analyses were conducted in Primer 6 (Primer-E Ltd, Plymouth, UK) using 999 re-runs to provide sufficient iterations for achieving convergence in the ordination solution. This resulted in four ordinations (spring and summer for both the coastal and offshore site). The resulting stress values were < 0.2, suggesting acceptable solutions [36]. Preliminary explorations revealed similar patterns of community dynamics during individual years. We therefore plotted averaged patterns for 5 periods, consisting of 4 years and the first of 2 years (1994–1995, 1996–1999, 2000–2003, 2004–2007, 2001–2008) to facilitate interpretation of the plots.

The NMDS analyses were followed by Spearman rank correlation analysis. Here, the raw biovolume data of each phytoplankton species were correlated with NMDS dimensions 1 and 2. We carried out separate analysis for each study year, using the ordination scores from the spring/summer and coastal/offshore NMDS ordinations and the phytoplankton biovolume raw data of these seasons/sites. This analysis facilitated an assessment of taxonomic change (frequencies and incidences of correlations of specific taxa) underlying the multivariate changes documented in the NMDS.

Permutational multivariate ANOVA (PERMANOVA) [37], a multivariate analogue to analysis of variance, was used as a numerical approach to quantify aspects of the adaptive cycle related to reorganization, conservatism and adaptation. PERMANOVA was carried out to contrast multivariate phytoplankton community structure between spring and summer blooms (B; i.e. average phytoplankton community structure in spring and summer; fixed factor), time (Ti; categorical factor comprising the study period between 1994 and 2011; random factor) and season nested in time (Se(Ti); covering the dynamic aspect within blooms; that is, three monthly values were used to characterize phytoplankton community dynamics during spring (March, April, May) and summer (June, July, August) respectively; random factor). Interactions between these terms were also tested in this model. Three terms were considered crucial for testing the predictions related to reorganization, conservatism and adaptation facets of phytoplankton adaptive cycles in the Baltic Sea: 1) The term “blooms (B)” allows for testing whether phytoplankton communities differ between spring and summer. From an adaptive cycle perspective it provides insight whether phytoplankton reorganize around a new structure, in terms of community composition, during summer after the spring cycle has collapsed. If reorganization occurs, this term will be significant. 2) The interaction term “B x Se(Ti)” tests for dynamic change of taxonomic composition within cycles. In the adaptive cycle context, these dynamics may be reminiscent of community build-up, saturation and collapse phases. Because these patterns are expected to be conservative, despite potential changes in phytoplankton community structure over the entire study period, no significant interaction term should be detected. 3) The interaction term “B x Ti” allows for testing whether species contributions to spring and summer phytoplankton community dynamics change over the study period. Given documented changes in phytoplankton community structure, particularly during summer found in other areas of the Baltic Sea during the period of our study [25–27] this term is expected to be significant.

PERMANOVA was calculated on a Bray–Curtis dissimilarity matrix of square-root transformed biovolume data of phytoplankton species. Because this analysis was unable to handle unbalanced designs (different temporal sampling resolution during seasons between years; see above), we averaged values to obtain three single-month values for each season (i.e. March, April, May for spring; June, July, August for summer), and using sites (coastal, offshore) as replicates in the analysis. Bray-Curtis dissimilarities allowed for assessing both the importance of species incidences and their abundances in the analyses. We contrasted the Bray-Curtis based approach, with a PERMANOVA analysis using data transformations into Sorensen dissimilarity matrices, which only accounts for changes in species presence/absence patterns. This allows assessing how sets of species change within adaptive cycles (spring, summer) and over the study period (1994–2011). We used 9999 unrestricted permutations of raw data using PERMANOVA v1.6 [37], and significance was assessed using Monte Carlo asymptotic P values. Because PERMANOVAs based on Bray-Curtis and Sorensen dissimilarity showed identical results, we show the results of the Bray-Curtis dissimilarity based results only.

## Results

### Water quality

**Fig 3:**
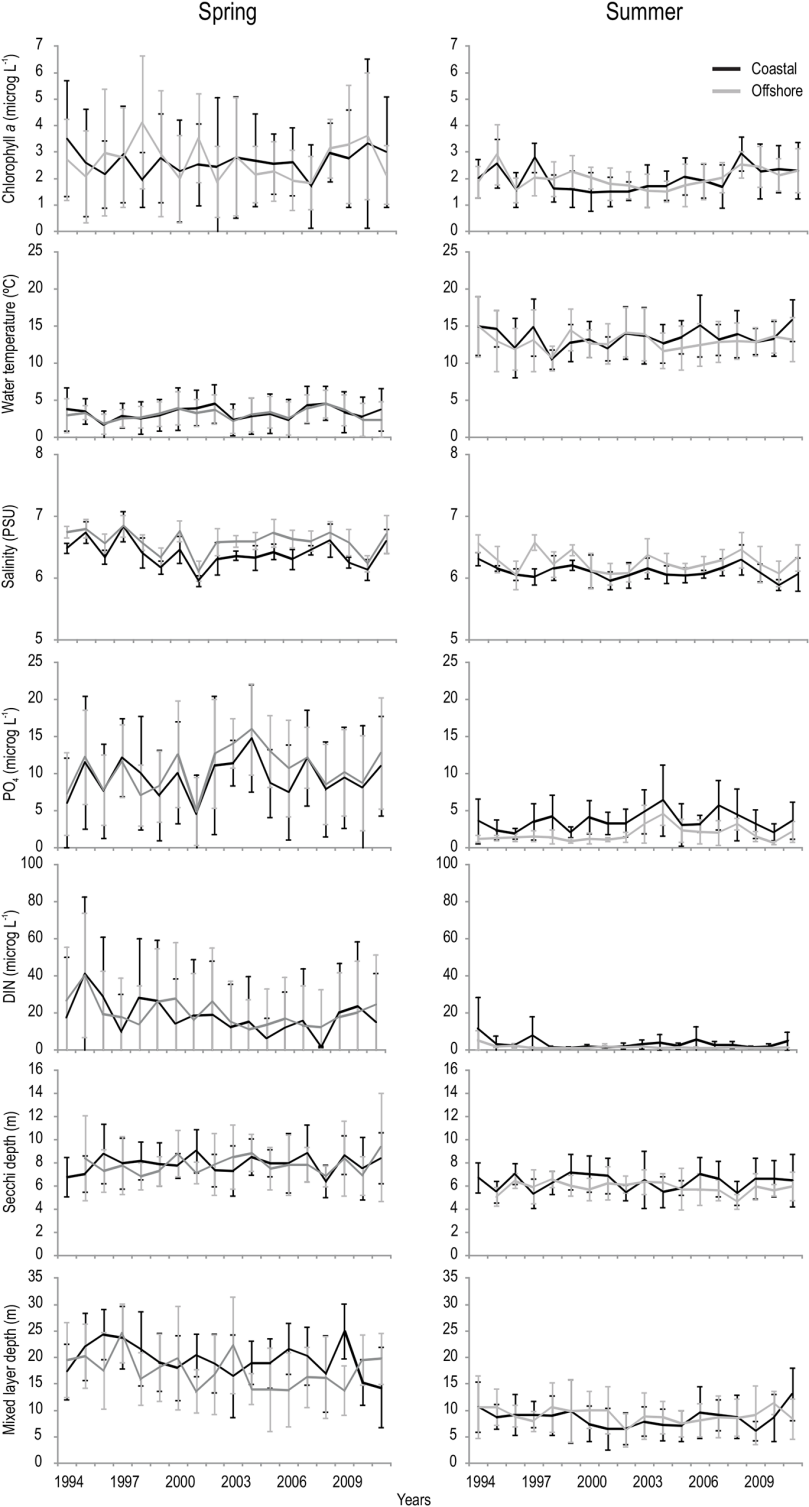
Temporal patterns of abiotic and biotic variables. Temporal patterns of phytoplankton biomass (Chlorophyll *a*) and abiotic variables at two stations (coastal, offshore) and seasons (spring, summer). Shown are seasonal averages ± SDs between 1994 and 2011.

**Fig 4:**
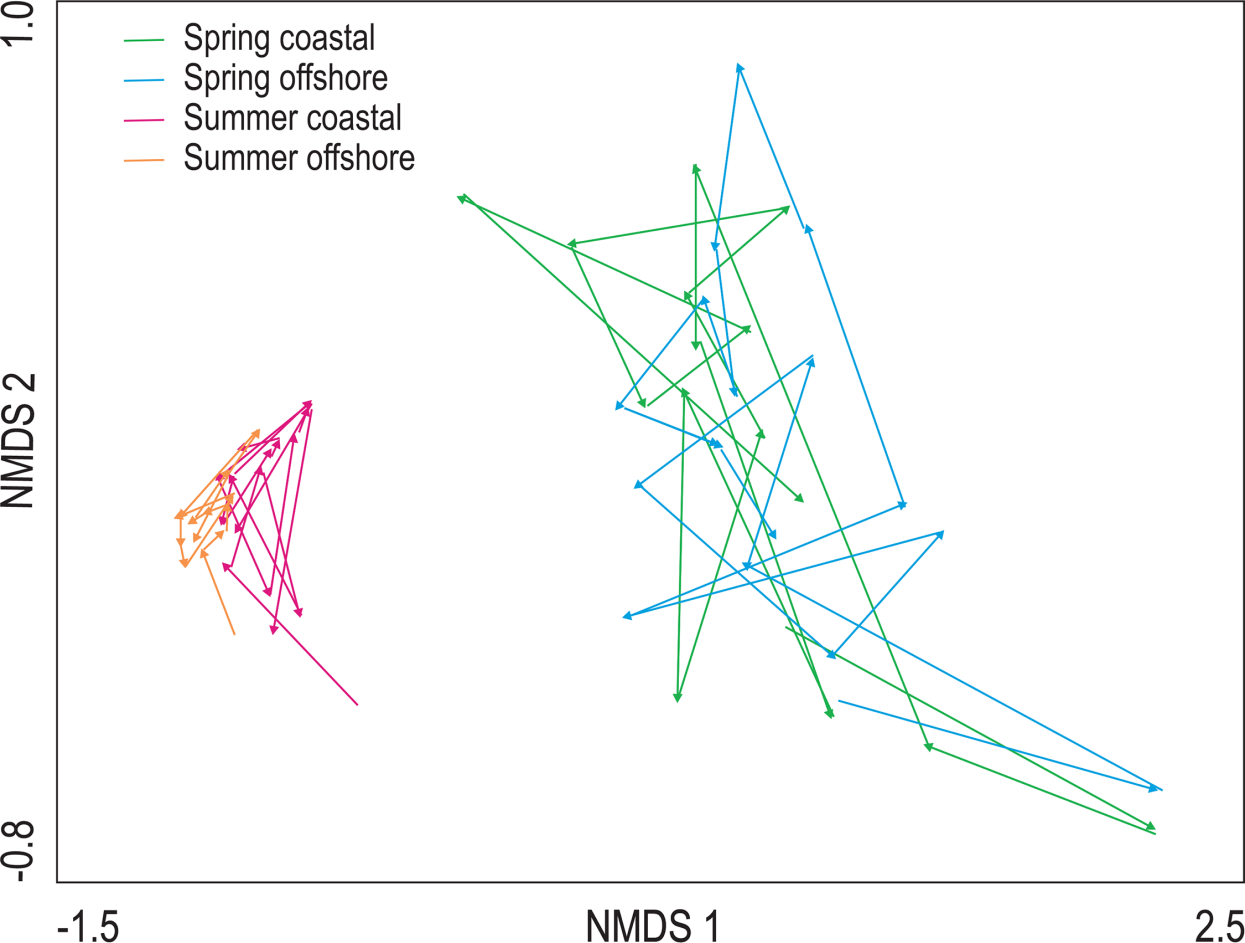
Multivariate ordination. Nonmetric multidimensional scaling (NMDS) ordinations showing seasonal averages of water quality trajectories during spring and summer in the coastal and offshore site between 1994 and 2011. The length of each arrow reflects change from one sampling year to the next.

The abiotic environment differed between both seasons at the coastal and offshore site, with average salinity, nutrients (phosphate, DIN), Secchi depth (indicating light conditions) and mixed layer depth being lowest in summer and water temperature being lowest in spring at both sites (Fig 3). Also phytoplankton biomass, measured as chlorophyll *a*, was lower in summer compared to spring at the coastal and offshore site (Fig 3). The differences in the abiotic conditions between seasons were also evident in the NMDS analysis, with spring and summer patterns clearly separated in ordination space (Fig 4). The between-year variability in water quality was higher in spring compared to summer, and both sites showed similar patterns of change in each season during the study period (Fig 4).

### Phytoplankton dynamics

PERMANOVA was used to quantify phytoplankton community dynamics in the Baltic Sea. The analysis detected a significant “bloom” (B) effect (Table 1), indicating that phytoplankton community structure differed between spring and summer blooms. The significant effect of Se(Ti) also captures the dynamic change of phytoplankton within each bloom, indicating distinct communities at the beginning and the end of each bloom. The significant effect of time (i.e. study years) highlights that the taxonomic composition of blooms is changing in the long term (1994–2011). The significant interaction term B x Ti highlights that phytoplankton community dynamics during spring and summer blooms differed over the study period, supporting the argument that spring and summer blooms differ in terms of community structure. Finally, the interaction term B x Se(Ti) was not significant. This indicates that, despite community changes observed in the long term, community trajectories within blooms are conservative over time.

**Table 1:** Results of PERMANOVA analysis contrasting multivariate community structure across blooms (B, phytoplankton spring and summer blooms), time (Ti, long-term period from 1994 to 2011), season nested in time (Se(Ti), 3 months comprising each bloom) and their interactions. Shown are degrees of freedom (df), sums of squares (SS), mean squares (MS), F-ratios (F), and the Monte Carlo asymptotic P values (P).

Source	df	SS	MS	F	P
B	1	74110	74110	31.2	0.0001
Ti	17	367770	2163	4.2	0.0001
Se(Ti)	36	18415	512	1.3	0.011
B x Ti	17	40425	2378	5.7	0.001
B x Se(Ti)	36	14973	416	1.1	0.223
Residual	108	40963	379		
Total	215	225656			

**Fig 5:**
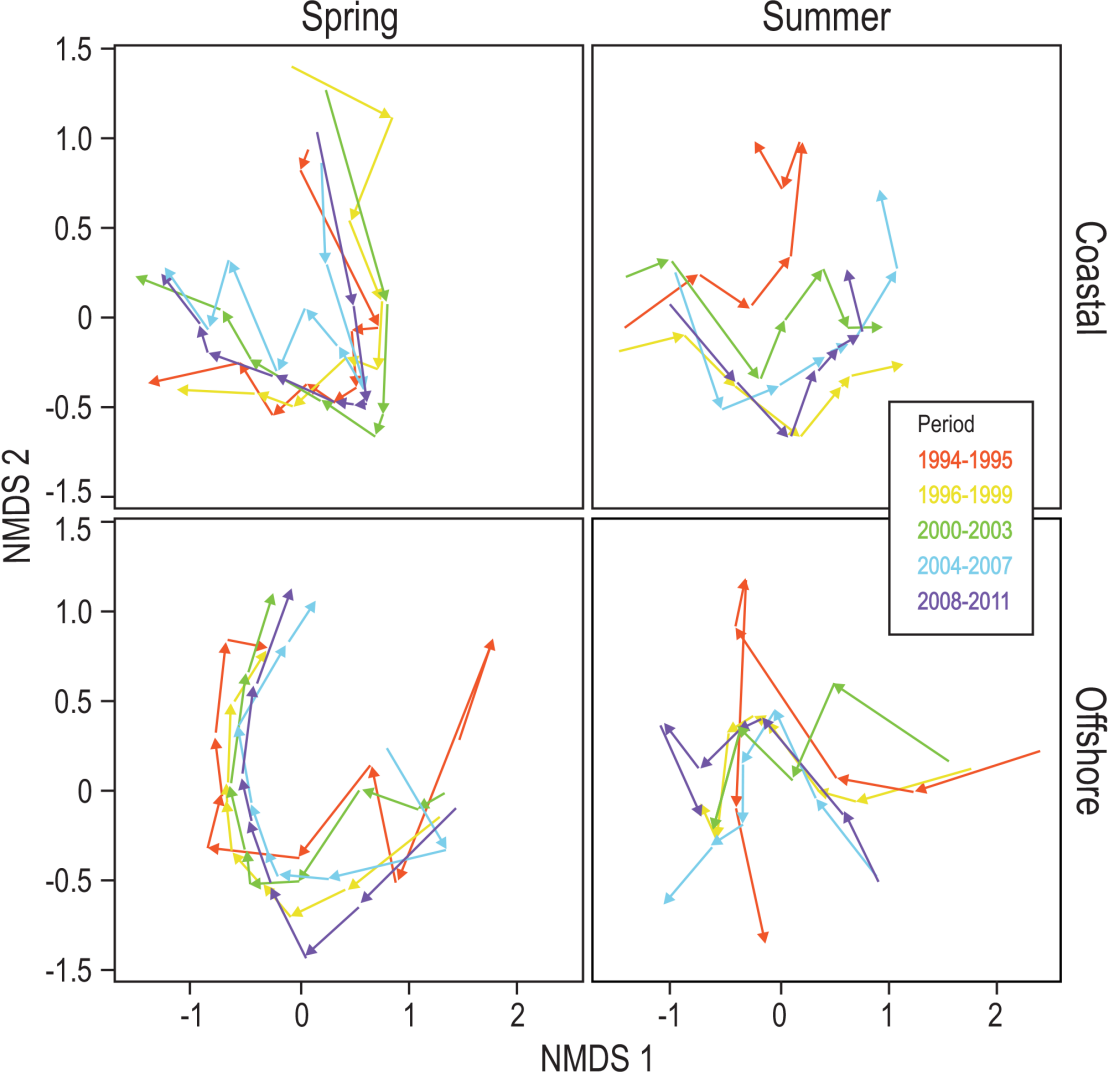
Multivariate ordination. Nonmetric multidimensional scaling (NMDS) ordinations showing community trajectories during spring and summer sampling occasions in the coastal and offshore site. Different colors represent averaged time periods. The length of each arrow reflects community change from one sampling event to the next.

The NMDS analysis underpins visually the results of the PERMANOVA. It revealed relatively conservative patterns of phytoplankton community dynamics during spring at the coastal and offshore sites over the study period (Fig 5), despite the differences in between-year variability observed for water quality (Fig 3). The movement in ordination space captured U-shaped trajectories in spring at both sites, highlighting distinct community structures during the beginning and end of spring that are reminiscent of the community build-up, saturation and collapse phases of the adaptive cycle model. During summer blooms the dynamics were more variable inter-annually at both sites relative to spring blooms. We observed both U-shaped patterns and zigzagging trajectories during distinct periods in summer (Fig 5). As was the case for spring dynamics, community composition at the beginning and end of summer differed, despite summer blooms showing more variable and irregular cycling behaviour relative to spring blooms.

**Fig 6:**
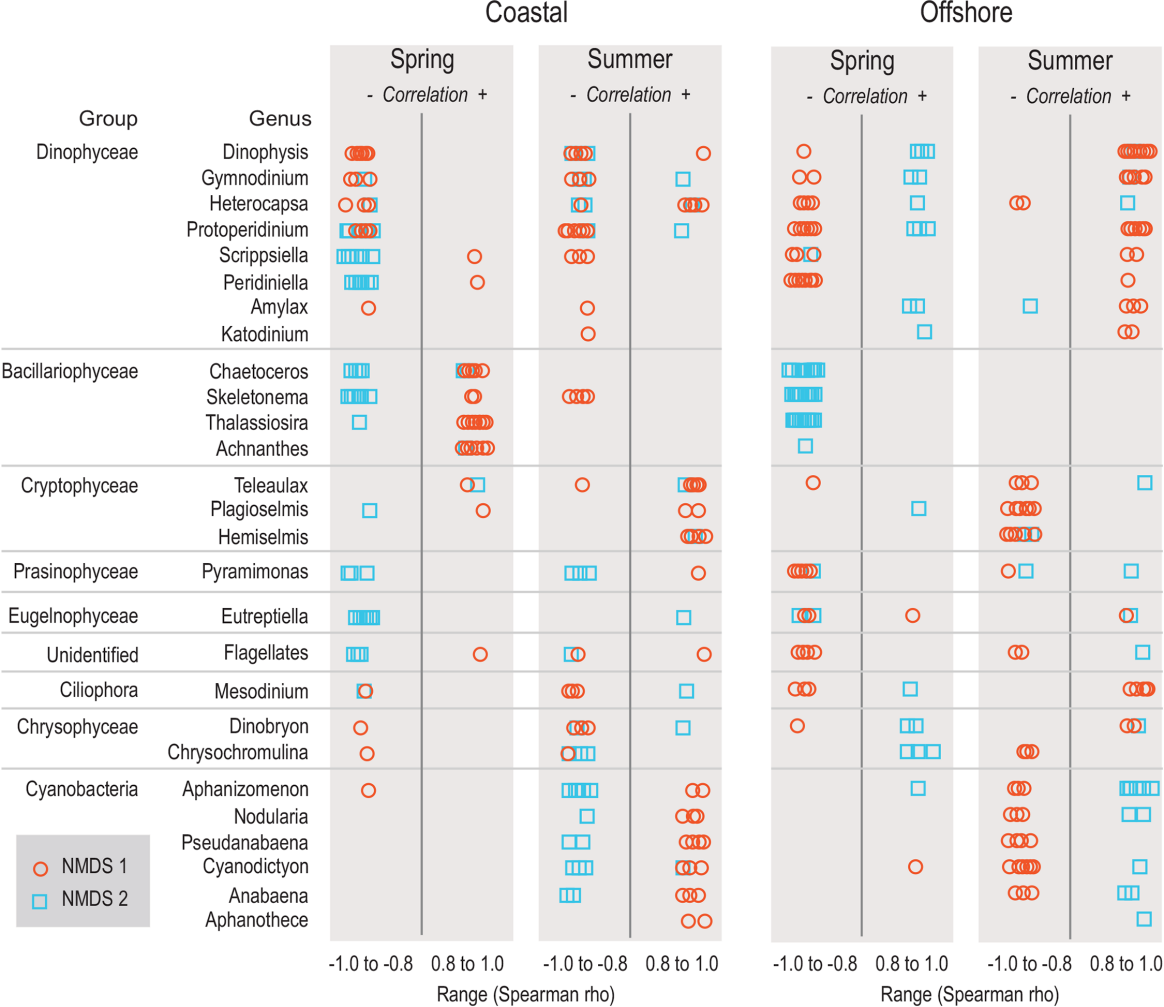
Species correlations with multivariate patterns. Score plots showing contributions of phytoplankton species to temporal trajectories of phytoplankton community dynamics identified by NMDS during spring and summer at the coastal and offshore site, based on Spearman rank correlation analyses. Only taxa with significant correlations with NMDS dimensions (P < 0.05) and high correlation coefficient (Spearman´s rho > 0.8) are shown. For better visibility taxa are aggregated to genus level.

Spearman rank correlation analysis revealed variability in the incidence of correlation of phytoplankton taxa with the NMDS dimensions over the study years (Fig 6). That is, positive correlations of species raw biovolume data with NMDS 1 and 2 dimensions indicate that a species dominates the phytoplankton community towards the right and upper part of the ordination, respectively. Negative correlations of species biovolumes with NMDS 1 and 2 highlight dominance towards the left and lower part of the ordination, respectively. For simplicity we only present species with high correlation coefficients (|0.8|—|1.0|), i.e. those dominating community dynamics described by the NMDS analysis. In practical terms we can describe several patterns that emerged from the analysis. First, during spring dynamics we observed broad shifts of communities from dominant diatoms (Bacillariophyceae), particularly *Thalassiosira* sp., *Skeletonema* sp. and *Chaetoceros* sp., to Dinophyta at both sites. Unidentified flagellates, *Pyramimonas* (Prasinophyceae), *Eutreptiella* (Euglenophyceae), and *Mesodinium* (Ciliophora) also contributed to these patterns, although to a different degree at both sites (Fig 6).

Cyanobacteria were generally not contributing to community dynamics during spring. However, during summer they showed a high incidence of correlations across study years, while diatoms played a little role in summer community trajectories (Fig 6). Dinophytes and *Mesodinium* were still important at the beginning of summer, highlighting “spill-over” from the spring cycles, but these taxa decreased over summer and were replaced by Cryptophyta, and occasionally Chrysophyceae.

## Discussion

In this study we used phytoplankton seasonal succession, a well-studied object in planktology, for evaluating ecological complexity theory, specifically facets of the adaptive cycle quantitatively. Results show that traditional phytoplankton successional models and the adaptive cycle are not mutually exclusive. The adaptive cycle offers a broader view that allows the concept of succession [2,3,22] to be neatly viewed within models of complex system dynamics [1,7].

Ecologists have a long-lasting interest in understanding patterns and processes influencing seasonal plankton dynamics [3,38]. Consistent with a multitude of studies in freshwater and marine environments, our study found a high turnover with species and major taxonomic groups replacing each other during the growing season. These taxonomic changes also entail functional changes in the phytoplankton communities. For instance, diatoms dominate spring blooms initially but were replaced by dinoflagellates towards the end of the spring bloom. Diatoms are fast growing when there is high nutrient availability and have therefore been referred to as r strategists by phytoplankton ecologists [39]. Dinoflagellates are slow growing, competitively inferior to diatoms and dominate during low nutrient concentrations [39]. They have been considered K strategists in phytoplankton ecology [40]. These examples show a good fit between phytoplankton life history strategies and phases of the adaptive cycle. Diatoms reflecting the r phase of the adaptive cycle and thus characterize community build-up, while dinoflagellates characterize the K phase of steady state conditions. This highlights that the adaptive cycle as a heuristic captures accurately ecological patterns in real ecosystems.

**Fig 7:**
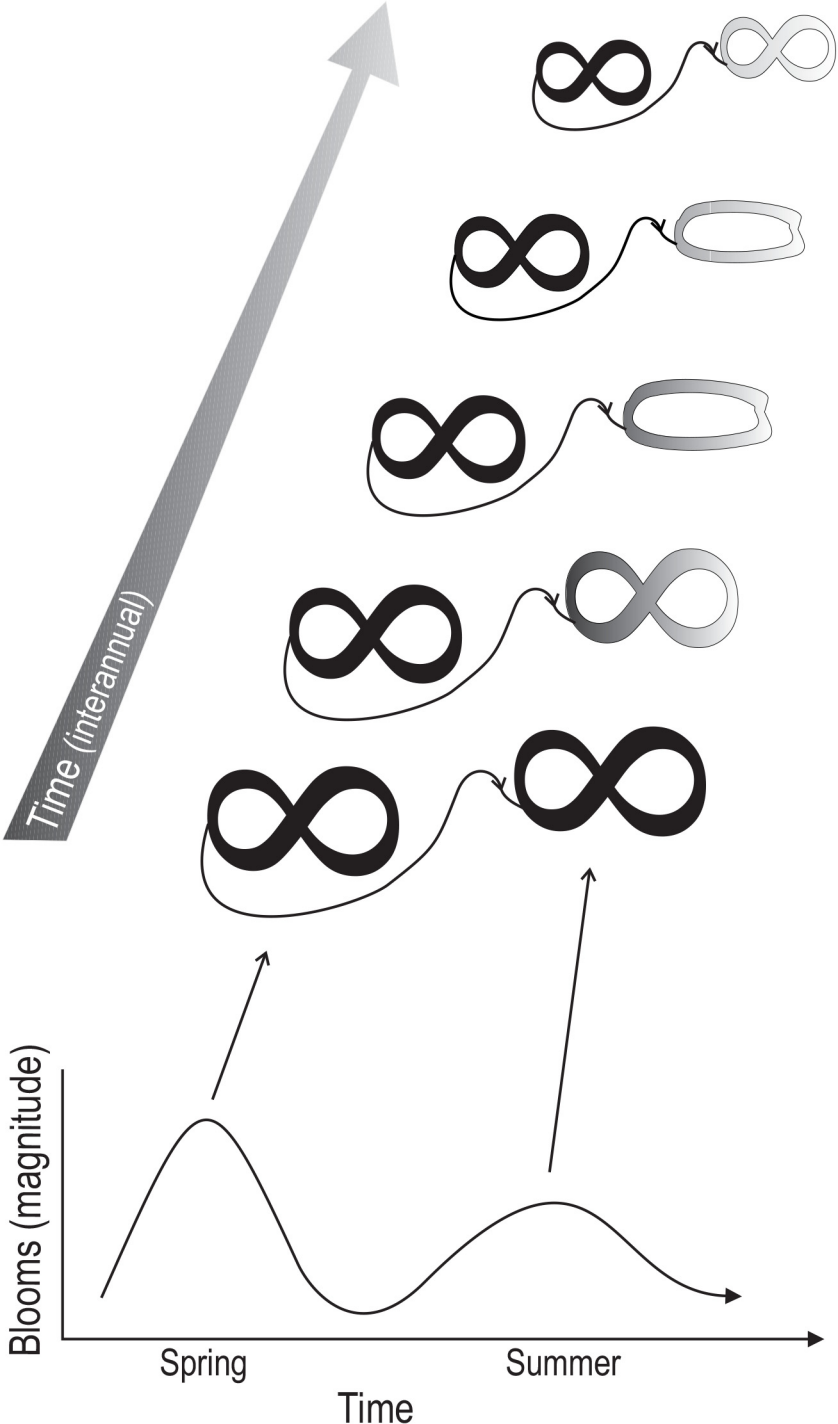
Conceptual figure. Summary of results of this study showing the complementarity of the traditional phytoplankton successional model (lower diagram) with adaptive cycles theory. Shown are spring and summer blooms and how they change interannually from an adaptive cycle perspective. Different shapes indicate that community changes within blooms are dynamic but these changes must not necessarily reflect adaptive cycle phases. These shapes of community change are often recurrent highlighting conservative patterns. Despite this, species contributions to these dynamics may change over time (different shades of gray), affecting spring and summer blooms distinctly, highlighting potential adaptive responses to environmental change.

Seasonal patterns in plankton communities have been generally discussed in the context of successional change [22]. By definition, ecological succession is the observed process of change in the species structure of an ecological community over time. The adaptive cycle provides a complementary view. The differences observed in phytoplankton community composition in spring and summer suggest a high turnover in phytoplankton community dynamics in the Baltic Sea, consistent with patterns documented for a plethora of marine systems and lakes [24,38]. This substantial ecological turnover suggests that spring and summer phytoplankton comprise alternative community states (Fig 7). This interpretation fits current theory that not only ecosystems but also ecological communities, which comprise complex adaptive systems [21], can undergo regime changes [1]. This community-level regime shift observed for phytoplankton is not only due to substantial abiotic change (temperature, salinity, nutrients, stratification) but also biotic change between spring and summer. This biotic change includes altered food web structure, resulting in higher zooplankton grazing and trophic cascades that influence community dynamics of phytoplankton [3,22,24,38]. This is reflected in the significant main effect “Blooms” in our PERMANOVA model, which found a significant difference in phytoplankton community composition between spring and summer blooms. Combined with the observation of fundamental changes in the environment, our findings suggest that the phytoplankton communities in spring and summer comprise alternative adaptive cycles, supporting our first prediction. This finding also supports a recent lake study, based on complex time series modelling, which has shown that phytoplankton seasonal succession across lakes with different disturbance regimes fits the adaptive cycle model [41].

In addition to the high turnover in community composition observed between spring and summer, our study and many others have found that even within blooms communities are highly dynamic, with community composition changing from the onset to the collapse of blooms. Also this within-bloom dynamics fit the adaptive cycle model well. The adaptive cycle as a simplified model of complex system dynamics emphasizes development through different stages (Figs 1 and 7). Some studies have suggested that these dynamics can be context dependent, and deviations from this simplified trajectory may occur in some systems [42–44]. This was also reflected in our study. Our results suggest that phytoplankton community dynamics can be conservative within a cycle but community composition can be plastic in the long-term. That is, the non-metric multidimensional scaling ordinations (NMDS) found similar spring dynamics at the coastal and offshore site over the study period, showing patterns reminiscent of community build-up, saturation and collapse phases of the adaptive cycle. These patterns were relatively stable over the study period, despite the high between-year variability in water quality. This variability can be ascribed to differences in environmental conditions during the beginning of blooms that in part depend on the severity and length of the winter season (O. Hjerne, unpublished results). On the other hand, the temporal patterns were less clear in summer, when greater variability, including oscillation in ordination space was observed. Despite this, the communities had a clear directional component of change, manifested in initial community structure developing towards a distinct community with different sets of species by the end of the bloom. This was reflected in our PERMANOVA results. The insignificant interaction term between Blooms and Season(Time) suggests that, despite the observed variability, within-bloom dynamics between spring and summer are similar. From an adaptive cycle perspective, this suggests that patterns of community change are conservative, which supports our second prediction.

In addition to assessing patterns of conservatism of community change observed during individual blooms, our study allowed us to explore community change over recurrent spring and summer blooms over the long term in the Baltic Sea. Specifically, phytoplankton blooms undergo taxonomic and phenological changes due to the interaction of global change stressors, including climate warming, eutrophication and fishing pressure [25–27]. The NMDS ordination demonstrated that patterns of change differ between summer and spring blooms. Largely overlapping temporal trajectories over the study period highlight similar taxonomic compositions during spring in the coastal and offshore site. In contrast, during summer, these patterns were more disparate, indicating that individual periods differed in their community composition and in their temporal trajectories.

We used time-averaged data in the NMDS for a simpler visual representation of community dynamics over the study period. Although between-year variability is compressed with this approach, our main aim was to assess the degree of similarity in community dynamics between sites (coastal, offshore) and season (spring, summer). The PERMANOVA results, which were based on the analyses of individual years, are in agreement with our NMDS ordinations, highlighting a significant interaction between Blooms and Time (i.e. study period 1994–2011). This highlights that the long-term dynamics of phytoplankton differ between spring and summer blooms, with spring blooms being taxonomically more stable over time relative to summer blooms. Such structural differences have been invoked previously with complex cause-effect chains between winter temperature and nutrient dynamics causing differential community responses in spring and summer [31]. This also supports our third prediction about adaptation in the long term. That is, environmental and biological filtering processes resulting from the interplay of natural and anthropogenic disturbances select for sets of species that are adapted to prevailing ecological conditions in the Baltic Sea in the long term. From a resilience perspective, this constant adjustment of ecosystem properties, including community composition, function and processes reflects a system level property, adaptive capacity, which focuses on “learning” and to anticipate and respond to disturbances [45,46].

We conclude by emphasizing the complementarity between “traditional” and “ecological complexity” approaches for describing patterns and processes in ecosystems. Common statistical methods and well-established ecological paradigms, including phytoplankton community seasonality, provide a significant advantage over complex modelling and simulation studies and simplified surrogacy/indicator approaches for describing the complexity inherent in ecological systems. Such an approach captures ecological patterns with high realism and allows for moving the adaptive cycle from a metaphor to a framework that can be empirically assessed. In turn, complexity theory, including the adaptive cycle model and its multi-scalar extension (Panarchy theory [1]), allows differentiating conservative patterns versus adaptation in complex systems dynamics undergoing change and has therefore strong potential to strengthen the nexus between ecological theory, environmental change and monitoring [47]. This may ultimately serve human societies for understanding how rapid environmental change affects the resilience of ecosystems [48].
